# Testing, diagnosis, and treatment following the implementation of a program to provide dried blood spot testing for HIV and hepatitis C infections: the NSW DBS Pilot

**DOI:** 10.1186/s12879-024-08989-8

**Published:** 2024-01-29

**Authors:** Nigel Carrington, Anna Conway, Jason Grebely, Mitchell Starr, Beth Catlett, Annabelle Stevens, Bianca Prain, Colette McGrath, Louise Causer, Rebecca Guy, Joanne Holden, Phillip Keen, Melanie Kingsland, Heng Lu, Cherie Power, Phillip Read, Carolyn Murray, Anna McNulty, Philip Cunningham, on behalf of the NSW DBS Pilot Study Group

**Affiliations:** 1 Centre for Population Health, NSW Ministry of Health, Sydney, Australia; 2 Sydney Sexual Health Centre, Sydney, Australia; 3 The Kirby Institute, UNSW, Sydney, Australia; 4 Centre for Social Research in Health, UNSW, Sydney, Australia; 5 NSW State Reference Laboratory for HIV, St Vincent’s Hospital, Sydney, Australia; 6 Vincent’s Centre for Applied Medical Research, St Vincent’s Hospital, Sydney, Australia; 7 NSW Justice Health & Forensic Mental Health Network, Sydney, Australia; 8 School of Medicine and Public Health, The University of Newcastle, Newcastle, Australia; 9 Population Health Research Program, Hunter Medical Research Institute, Newcastle, Australia; 10 Hunter New England Population Health, Hunter New England Local Health District, Newcastle, Australia; 11 Kirketon Road Centre, South Eastern Sydney Local Health District, Sydney, Australia; 12 School of Population Health, UNSW, Sydney, Australia

**Keywords:** HIV, Hepatitis C, Dried blood spot, Testing, Implementation, Models of care

## Abstract

**Background:**

Dried blood spot (DBS) testing provides an alternative to phlebotomy and addresses barriers to accessing healthcare experienced by some key populations. Large-scale evaluations of DBS testing programs are needed to understand their feasibility. This study evaluated the implementation of a state-wide DBS HIV and hepatitis C virus (HCV) testing pilot.

**Methods:**

The New South Wales (NSW) DBS Pilot is an interventional cohort study of people testing for HIV antibody and/or HCV RNA from DBS samples in NSW, Australia. Participants at risk of HIV/HCV participated in testing via: 1) self-registration online with a DBS collection kit delivered and returned by conventional postal service; or 2) assisted DBS sample collection at 36 community health sites (including drug treatment and harm-minimisation services) and prisons. Participants received results by text (HIV antibody/ HCV RNA not detected) or a healthcare provider (HIV antibody/ HCV RNA detected). The RE-AIM framework was used to evaluate reach, effectiveness, adoption, and implementation.

**Results:**

Reach: Between November 2016 and December 2020, 7,392 individuals were tested for HIV and/or HCV (21% self-registration, 34% assisted in community, and 45% assisted in prison). Effectiveness: Of 6,922 people tested for HIV (19% men who have sex with men, 13% living outside major cities, 21% born outside Australia), 51% (3,521/6,922) had no HIV test in the past two years, 0.1% (10/6,922) were newly diagnosed with HIV, and 80% (8/10) initiated HIV treatment within six months. Of 5,960 people tested for HCV (24% women, 35% Aboriginal and/or Torres Strait Islander, 55% recently injected drugs), 15% had detectable HCV RNA (878/5,960), and 45% (393/878) initiated treatment within six months. Adoption: By the end of 2020, DBS via assisted registration was available at 36 community sites and 21 prisons. Implementation: 90% of DBS cards arriving at the laboratory had the three full spots required for testing; the proportion was higher in assisted (94%) compared to online (76%) registration.

**Conclusions:**

This study demonstrated the feasibility of DBS testing for HIV and HCV in key populations including Aboriginal and Torres Strait Islander peoples, men who have sex with men, people who inject drugs, and demonstrated the utility of DBS in the prison setting.

**Supplementary Information:**

The online version contains supplementary material available at 10.1186/s12879-024-08989-8.

## Background

Stopping HIV transmission and eliminating hepatitis C virus (HCV) infection are key aims of global and national health strategies [1–3]. Home-based self-testing improves HIV testing uptake [4] and dried blood spot (DBS) sampling improves HCV testing uptake [5]. Few studies have evaluated the large-scale implementation of DBS as a strategy to improve HIV and HCV testing uptake among key populations.

Traditional testing pathways for HIV and HCV can be complex and involve multiple visits which may delay diagnosis and access to care or increase loss to follow-up [6–8]. For HCV, the traditional testing pathway involves an HCV antibody test to confirm exposure and, among people who are HCV antibody positive, an HCV RNA test to detect current infection. This two-step pathway often requires multiple visits to practitioners and off-site phlebotomists, leading to many people who are HCV antibody-positive never receiving confirmatory HCV RNA testing and thus linkage to care [9, 10]. For people who have a long history of injecting drug use, poor venous access can pose challenges for sample collection [11], leading to negative experiences of blood sample collection which may present a barrier for future HIV and HCV testing [11].

DBS sampling enables home self-collection or assisted collection at trusted services and requires minimal resources for collecting, storing and transporting blood specimens, making it suitable in many settings [12]. Fingerstick samples reduce the need for venepuncture, making it more accessible for people with poor venous access [13]. DBS testing has been shown to improve HCV testing uptake compared to standard of care [5, 14, 15] but may be inferior to rapid point-of-care testing in terms of treatment initiation [16, 17]. Large-scale evaluations of DBS testing programs are critical to understand feasibility, acceptability, and impact to inform health service planning.

The RE-AIM framework has been developed to facilitate research translation into practice [18]. The framework uses five dimensions to understand the public health benefit of an intervention, including those at an individual level (Reach, Effectiveness, and Maintenance) and staff/setting levels (Adoption, Implementation, Maintenance) [18]. The framework can be applied flexibly and adapted to the available evaluation resources through “pragmatic use of key dimensions rather than comprehensive applications of all elements” [18] and in this study we report on four dimensions. This study used the RE-AIM framework to evaluate the reach, effectiveness, adoption, and implementation of DBS sampling for HIV and HCV testing in New South Wales, Australia.

## Methods

### Study population and design

The NSW DBS Pilot is an observational cohort study of participants at risk of HIV or HCV infection in New South Wales, Australia. Participants were recruited via online self-registration for home-based sample collection, assisted registration at community sites (drug treatment, needle and syringe programs, mobile outreach, homelessness settings, or mental health settings) or assisted registration in prison. DBS sampling was used to test for HIV antibody and/or HCV RNA. Online self-registration was primarily targeted to people at risk of HIV while assisted registration pathways was primarily targeted people at risk of HCV. Only DBS cards which provided three full spots of blood sample were able to be tested and included in the analysis. Participants for this analysis were enrolled between November 2016 and December 2020 and followed until June 30, 2021 (six-months post-testing).

The NSW DBS Pilot began in November 2016, offering HIV testing via online self-registration to people aged ≥ 16 years self-reporting the following HIV risk factors: gay and other men who have sex with men (MSM), people from Sub-Saharan Africa and Southeast Asia, and people with current/previous sexual partners from Sub-Saharan Africa and Southeast Asia. In September 2017, assisted site-based registration and HCV testing were introduced, with Aboriginal or Torres Strait Islander people and people with a history of injecting drug use eligible for inclusion. Following the introduction of HCV testing, people eligible for HCV testing were automatically eligible for HIV testing; participants were asked if they wanted testing for HCV alone or HCV and HIV. In June 2019, inclusion criteria for HCV testing were expanded to include people born in Asia/Africa and people with a history of incarceration.

The study protocol and amendments were approved by St Vincent’s Hospital (Sydney) Human Research Ethics Committee (2019/ETH09614 HREC/15/SVH/400), the Aboriginal Health and Medical Research Council Human Research Ethics Committee, and the NSW Corrective Services Ethics Committee.

### DBS testing

DBS collection kits distributed to sites and study participants included a test card, a lancet, alcohol swabs, band-aids, cotton balls, a foil envelope, and a paid return envelope. From December 2017, kits included a visual aid to facilitate sample collection (Supplementary Fig. 1). Procedures for DBS sample collection and testing (elution, spot size, punching protocols, validation, testing algorithm) are described elsewhere [19, 20]. Tests used in the NSW DBS Pilot included: Murex HIV-1.2.0 antibody ELISA (Diasorin, Macquarie Park, Australia) for HIV antibody, New Lav-Blot-1 (Bio-Rad, Gladesville, Australia) and Aptima HCV Quant Dx assay (Hologic, Macquarie Park, Australia) for HCV RNA testing. The testing algorithm is shown in Supplementary Fig. 2 [19, 21, 22]. All detectable results were confirmed with testing via venepuncture.

### Procedures

The study methods have been described previously [23]. For online registration, participants accessed a public website (https://dbstest.health.nsw.gov.au/) to assess eligibility, received a collection kit, and delivered a sample by return paid envelope to be tested at the St Vincent’s Centre for Applied Medical Research Centre, NSW State Reference Laboratory for HIV, St Vincent’s Hospital, Sydney. The online survey was available in Chinese (Traditional), Indonesian, Thai, Vietnamese, Arabic, Portuguese, French and Spanish (community languages recommended by Multicultural HIV and Hepatitis Service).

For assisted registration in community, the online survey was completed with the assistance of site staff and/or peer workers who then collected a DBS sample. Samples were returned to the laboratory.

Assisted registration in prison was primarily offered through high-intensity testing campaigns run by Justice Health (a service of the state department of health and separate to corrective services) over 2–3 days at one site. One of the inclusion criteria for testing is ever being in prison, therefore anyone currently in prison was eligible for the study. The testing was advertised in advance (where possible, in collaboration with committees of people who are incarcerated in that prison) and sample collection stations were set up in recreational areas within the prison to avoid the need for people to be escorted to and from the prison health centre for sample collection. External staff with lived experience, such as peer workers from community-led organisations (NSW Users and AIDS Association and Hepatitis NSW) were involved in the recruitment of participants, to explain what the study involved and to explain possibilities for treatment. The survey was completed with the assistance of Justice Health staff who then collected a DBS sample. Samples were returned to the laboratory.

Results were provided via SMS, phone call, or site staff, depending on recruitment setting, linkage to confirmatory testing and care was provided either by the site or the state-wide sexual health service. All sites completed a case report form for people with a non-negative result which provided information on treatment initiation or loss to follow-up at six months.

No study-specific compensation was offered, but some sites implemented incentives through local initiatives.

### Implementation support to sites

Sites were assisted by a state-wide coordinator to obtain ethics approval, apply for local governance approval and set up their investigator site files. Site staff were provided training in person or virtually and were guided to develop standard operating procedures and to determine a suitable care pathway for participants. The state-wide coordinator liaised between sites and the testing laboratory to order test kits and to manage any problems with test registrations or sample quality. All costs for test kits, pathology services, training and support were borne by the sponsor (not the site or participant).

### Exposures

Demographic and behavioural factors hypothesised to be associated with no recent testing were determined from the literature and included: (i) testing setting (online self-registration, assisted registration in community, assisted registration in prison) (ii) gender [male, female, other (including non-binary and transgender)], (iii) age at survey, (iv) Aboriginal and/or Torres Strait Islander, (v) born outside of Australia [no, yes (Asia/Africa), yes (other)], (vi) speaks English at home, (vii) recently injected drugs (no, yes, prefer not to say). Due to changes in the survey from the beginning of 2019, the definition of recent drug injection changed from ‘in last 12 months’ (pre-2019) to ‘in last month’ (2019 onwards).

### Statistical analysis

This study used the RE-AIM dimensions to evaluate the following measures:

#### Reach

The number of tests performed over the study period were assessed by registration pathway and test type (HIV/HCV/both).

#### Effectiveness

The proportion of people with no recent (last two years) HIV or HCV test was assessed among people tested for HIV or HCV, respectively. Logistic regression models were used to estimate crude and adjusted odds ratios (aOR) and corresponding 95% confidence intervals (95% CI) to evaluate the factors associated with no recent HIV/HCV testing. Variables with *p* value < 0.10 in the unadjusted logistic regression models were retained in adjusted models if no collinearity was identified.

The proportion of people newly diagnosed with HIV, the proportion of people with detectable HCV RNA, and proportion initiating treatment for HIV and HCV was assessed.

#### Adoption

The number and type of sites participating in the assisted registration pathway and the number of tests per site per year were assessed.

#### Implementation

The proportion of all returned samples with adequate spots for testing was assessed. The median time from registration to sample testing was assessed overall and by registration pathway.

For all analyses, statistically significant differences were assessed at a 0.05 level; *p*-values were two-sided. All analyses were performed using Stata v14.0 (StataCorp, College Station, Texas).

## Results

### Reach

**Fig. 1:**
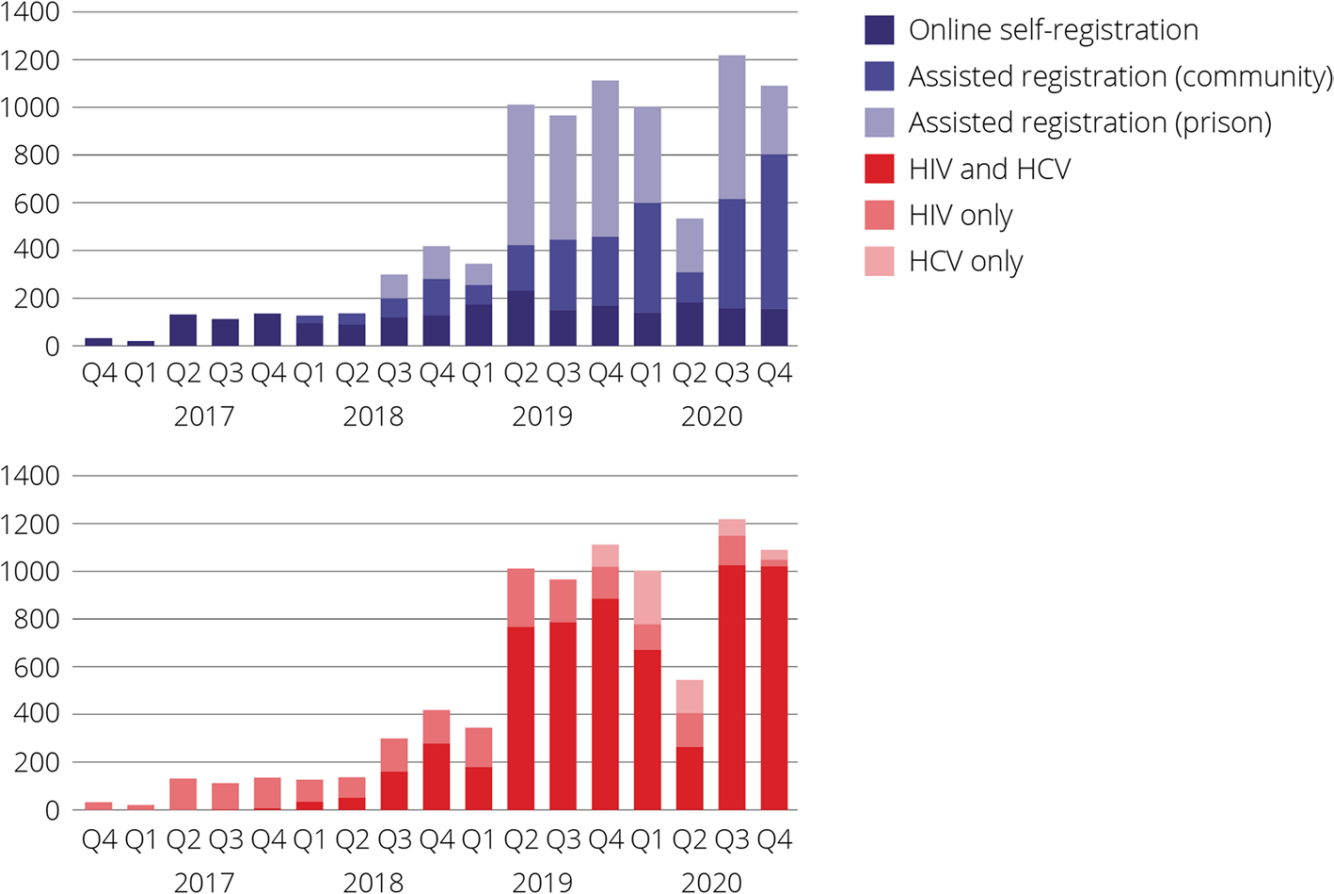
People tested within the NSW DBS Pilot, by registration pathway and analyte tested

Between November 2016 and December 2020, there were 8696 tests among 7392 people. Overall, 21% (1559/7392) participated via online self-registration, 34% (2523/7392) via assisted registration in the community, and 45% (3310/7392) via assisted registration in prison (Table 1). The number of people tested increased between 2017 and 2020 (430, 2017; 979, 2018; 3433, 2019; and 3854, 2020). Online self-registration testing stayed constant, while assisted registration testing increased during the study period, with a decline in testing observed in Quarter 2 of 2020 following the beginning of the COVID-19 pandemic (Fig. 1). Most people were tested for HIV and HCV (74%, Fig. 1).

**Table 1:** Characteristics of people tested by registration type (NSW DBS Pilot, November 2016- December 2020)

	People tested		Online self-registration		Assisted registration community		Assisted registration prison		
	N	%	n	%	n	%	n	%	*p*
Total	7392		1559		2523		3310		
Test performed									
HIV + HCV	5467	74%	269	17%	2275	90%	2923	88%	
HIV only	1441	19%	1282	82%	123	5%	36	1%	
HCV only	484	7%	8	1%	125	5%	351	11%	< 0.001
Gender									
Male	5752	78%	1340	86%	1626	64%	2786	84%	
Female	1573	21%	205	13%	855	34%	513	15%	
Other	67	1%	14	1%	42	2%	11	0%	< 0.001
Age									
= < 25	1194	16%	490	31%	152	6%	552	17%	
25–34	2308	31%	607	39%	394	16%	1307	39%	
35–44	1981	27%	243	16%	832	33%	906	27%	
45–54	1279	17%	120	8%	760	30%	399	12%	
> 55	630	9%	99	6%	385	15%	146	4%	< 0.001
Aboriginal and/or Torres Strait Islander									
No	5255	71%	1499	96%	1791	71%	1965	59%	
Yes	2137	29%	60	4%	732	29%	1345	41%	< 0.001
Major city postcode^a^									
No	894	21%	354	23%	540	21%			
Yes	3188	78%	1205	77%	1983	79%			0.328
Men who have sex with men									
No	6054	82%	548	35%	2287	91%	3219	97%	
Yes	1338	18%	1011	65%	236	9%	91	3%	< 0.001
Sexual partner from Asia or Africa									
No	6171	83%	981	63%	2300	91%	2890	87%	
Yes	1221	17%	578	37%	223	9%	420	13%	< 0.001
Born outside of Australia									
No	5896	80%	845	54%	2145	85%	2906	88%	
Yes, Asia or Africa	757	10%	489	31%	108	4%	160	5%	
Yes, other	739	10%	225	14%	270	11%	244	7%	< 0.001
Speaks English at home									
No	813	11%	418	27%	142	6%	253	8%	
Yes	6579	89%	1141	73%	2381	94%	3057	92%	< 0.001
Recently injected drugs									
No	3780	51%	1436	92%	756	30%	1588	48%	
Yes	3360	45%	98	6%	1655	66%	1607	49%	
Prefer not to say	250	3%	23	1%	112	4%	115	3%	< 0.001

Proportions are column proportions

*Acronyms*: *HCV* Hepatitis C virus

^a^Major city postcode not reported for prison setting

Compared to assisted registration in community/prison, the online self-registration pathway reached a higher proportion of people aged < 25, MSM, people with a sexual partner from Asia or Africa, people born in Asia or Africa and people who do not speak English at home (Table 1). Assisted registration in community settings reached a higher proportion of people who recently injected drugs. Assisted registration in prison reached a higher proportion of Aboriginal and Torres Strait Islander people. For both assisted registration in the community and the online self-registration pathway, around a fifth of participants lived outside of major cities.

### Effectiveness

Among people tested for HIV (*n* = 6922), 51% had not been tested for HIV in the two years prior to enrolment. Factors associated with not having received a recent HIV test included older age (> 55 vs < 25 years, aOR 1.37; 95%CI 1.11–1.70) and speaking a language other than English at home (aOR 1.28; 95%CI 1.09–1.50). People who were Aboriginal or Torres Strait Islander (aOR 0.75; 95%CI 0.67–0.84), men who have sex with men (aOR 0.67; 95%CI 0.58–0.76), and people who had recently injected drugs (aOR 0.51; 95%CI 0.46–0.56) had a reduced odds of not having received recent HIV testing (Table 2).

**Table 2:** Characteristics of people not recently tested for HIV by registration type (NSW DBS Pilot November 2016- December 2020)

	Total people tested for HIV in pilot	No HIV test in last two years		Factors associated with no HIV test in last two years	
	N	n	%	cOR	aOR
Total	6922	3521	51%		
Registration pathway					
Online self-registration	1551	931	60%	1.35 (1.19–1.54)	
Assisted registration—community	2401	1264	53%	1	
Assisted registration—prison	2970	1326	45%	0.73 (0.65–0.81)	
Test performed					
HIV + HCV	5481	2676	49%		
HIV only	1441	845	59%		
Gender					
Male	5365	2724	51%	1	
Female	1491	767	51%	1.03 (0.92–1.15)	
Other	66	30	45%	0.81 (0.50–1.32)	
Age					
= < 25	1138	662	58%	1	1
25–34	2158	1016	47%	0.64 (0.55–0.74)	0.64 (0.56–0.75)
35–44	1851	833	45%	0.59 (0.51–0.68)	0.65 (0.55–0.76)
45–54	1195	629	53%	0.80 (0.68–0.94)	0.90 (0.76–1.07)
> 55	580	381	66%	1.38 (1.12–1.69)	1.37 (1.11–1.70)
Aboriginal and/or Torres Strait Islander					
No	4953	2650	54%	1	1
Yes	1969	871	44%	0.69 (0.62–0.77)	0.75 (0.67–0.84)
Men who have sex with men					
No	5599	2859	51%	1	1
Yes	1323	662	50%	0.96 (0.85–1.08)	0.67 (0.58–0.76)
Sexual partner from Asia or Africa					
No	5729	2810	49%	1	
Yes	1193	711	60%	1.53 (1.35–1.74)	
Born outside of Australia					
No	5482	2658	48%	1	
Yes, Asia or Africa	741	468	63%	1.82 (1.55–2.13)	
Yes, other	699	395	57%	1.38 (1.18–1.62)	
Speaks English at home					
No	788	485	62%	1.63 (1.40–1.90)	1.28 (1.09–1.50)
Yes	6134	3036	49%	1	1
Recently injected drugs					
No	3477	2027	58%	1	1
Yes	3203	1338	42%	0.51 (0.47–0.57)	0.51 (0.46–0.56)
Prefer not to say	240	155	65%	1.30 (0.99–1.71)	1.23 (0.93–1.62)

Proportions are row proportions

*Acronyms*: *HCV* hepatitis C virus, *cOR* crude Odds Ratio, *aOR* adjusted Odds Ratio

Among 6922 people tested for HIV, 10 people were newly diagnosed (0.1%) (*n* = 9 via online registration; *n* = 1 in prison). Eight (8/10, 80%) initiated treatment within six months (one initiated treatment after six months and one returned to their home country).

Of all people tested for HCV with available data (*n* = 4112), 45% had not been tested in the two years prior to enrolment. Factors associated with not having received a recent HCV test included older age (> 55 vs < 25 years, aOR 1.47; 95%CI 1.12–1.93) and not speaking English at home (aOR 1.59; 95%CI 1.25–2.02). People who were Aboriginal or Torres Strait Islander (aOR 0.76; 95%CI 0.66–0.88) and had recently injected drugs (aOR 0.46; 95%CI 0.40–0.53) had a reduced odds of not having received recent HCV testing (Table 3).

**Table 3:** Characteristics of people not recently tested for HCV RNA by registration type (NSW DBS Pilot September 2019^a^- December 2020)

Variables	Total people tested for HCV in pilot	No HCV test in last two years		Factors associated with no HCV test in last two years	
	N	n	%	cOR	aOR
Total	4112	1851	45%		
Registration pathway					
Online self-registration	240	177	74%	3.58 (2.65–4.84)	
Assisted registration—community	1815	798	44%	1	
Assisted registration—prison	2057	876	43%	0.95 (0.83–1.07)	
Test performed					
HIV + HCV	3581	1675	47%		
HCV only	531	176	33%		
Gender					
Male	3150	1433	45%	1	
Female	918	394	43%	0.90 (0.78–1.04)	
Other	44	24	55%	1.44 (0.79–2.61)	
Age					
= < 25	495	245	49%	1	1
25–34	1192	500	42%	0.74 (0.60–0.91)	0.76 (0.61–0.94)
35–44	1212	466	38%	0.64 (0.52–0.79)	0.73 (0.58–0.90)
45–54	806	400	50%	1.01 (0.80–1.26)	1.20 (0.95–1.52)
> 55	407	240	59%	1.47 (1.13–1.91)	1.47 (1.12–1.93)
Aboriginal and/or Torres Strait Islander					
No	2755	1333	48%	1	1
Yes	1357	518	38%	0.66 (0.58–0.75)	0.76 (0.66–0.88)
Men who have sex with men					
No	3824	1701	44%	1	1
Yes	288	150	52%	1.36 (1.07–1.72)	1.28 (1.00–1.65)
Born outside of Australia					
No	3430	1439	42%	1	
Yes, Asia or Africa	284	185	65%	2.59 (2.01–3.33)	
Yes, other	398	227	57%	1.84 (1.49–2.27)	
Speaks English at home					
No	337	210	62%	2.15 (1.71–2.71)	1.59 (1.25–2.02)
Yes	3775	1641	43%	1	1
Recently injected drugs					
No	2406	1233	51%	1	1
Yes	1481	474	32%	0.45 (0.39–0.51)	0.46 (0.40–0.53)
Prefer not to say	225	144	64%	1.69 (1.27–2.25)	1.74 (1.30–2.32)

Proportions are row proportions

*Acronyms*: *HCV* hepatitis C virus, *cOR* crude Odds Ratio, *aOR* adjusted Odds Ratio

^a^Question on HCV testing recency was introduced in September 2019

Among people tested for HCV, 15% (878/5960) had detectable HCV RNA; 5% (15/328) in online self-registration, 17% (409/2357) in assisted registration in community, 14% (454/3275) in assisted registration in prison (*p* < 0.001). Among people with detectable HCV RNA, 45% (393/878) initiated treatment within six months;13% (2/15) in online self-registration, 26% (107/409) in assisted registration in community, 61% (277/454) in assisted registration in prison (*p* < 0.001).

### Adoption

By the end of 2020, DBS via assisted registration was available at 36 community sites and 21 prisons. Among community sites, the median time from study enrolment to the end of the study period was 2.3 years (IQR 1.7–2.7 years) and the median number of people tested per site per year was 18 (IQR 5–46).

### Implementation

**Fig. 2:**
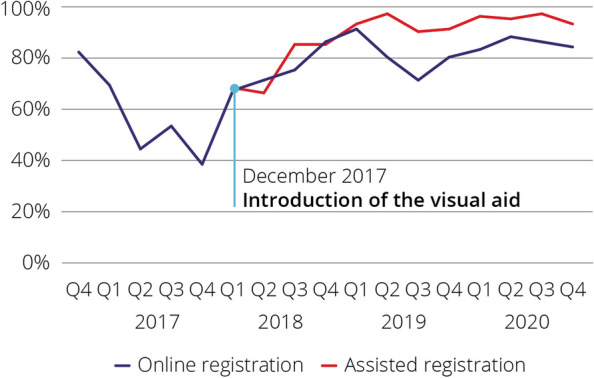
Proportion of DBS cards returned with three full spots by quarter and registration type (NSW DBS Pilot November 2016- December 2020)

Overall, 90% of DBS cards arriving at the laboratory had three full spots required for testing, with a higher proportion in assisted (94%) compared to online testing (76%, *p* < 0.001) (Fig. 2). In December 2017, a visual aid to support sample collection was introduced (Supplementary Fig. 1), leading to a significant increase in the proportion of cards with three full spots among those receiving online testing (51% from November 2016–December 2017 vs 82% 2018–2020, *p* < 0.001) (Fig. 2).

**Fig. 3:**
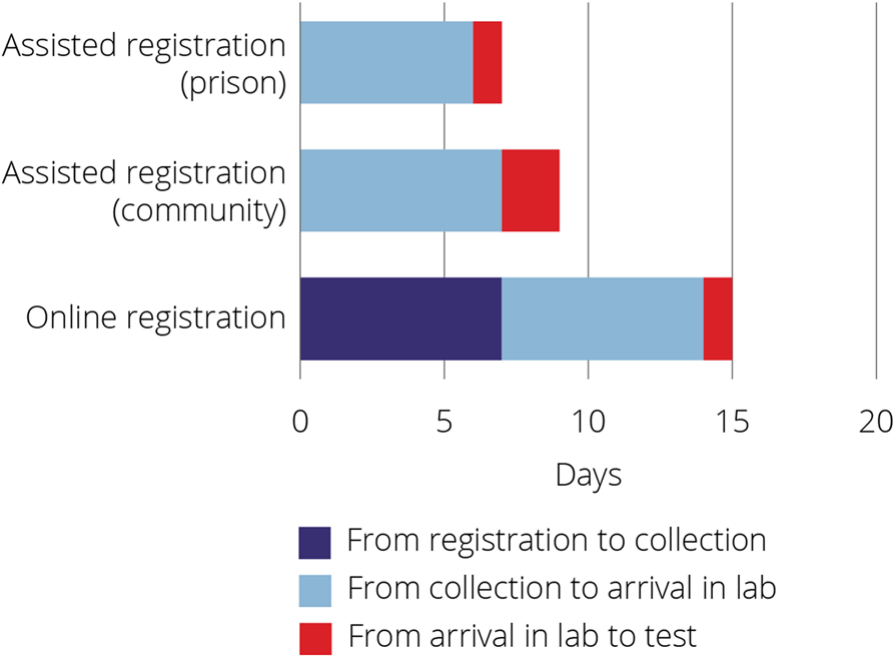
Median number of days of each stage of DBS testing by registration type (NSW DBS Pilot November 2016- December 2020)

The median time from registration to lab arrival was 7 days for assisted registration in prison, 9 days for assisted registration in community, and 15 days for online self-registration (Fig. 3).

## Discussion

This study applied the RE-AIM framework to evaluate the reach, effectiveness, adoption, and implementation of a state-wide program to enhance HIV and HCV testing through DBS sample collection in New South Wales, Australia. DBS facilitated increased reach of testing, particularly for certain sub-groups such as those who had not recently tested. Testing for HIV via online self-registration had high uptake among MSM and people born overseas. Assisted registration with DBS was shown to be valuable in detecting current HCV infection in both the community and prison settings, particularly among people who inject drugs. Minimal operator training and its suitability for use outside of clinical settings means that DBS for HIV and HCV testing is a useful tool in the home, community, and prison.

The overall HIV prevalence (0.1%) is lower than that among gay and bisexual men attending publicly funded sexual health clinics and private GP clinics with high caseloads of gay and bisexual men in NSW (0.5% in 2022 [24]). The online self-registration pathway was most successful at testing key populations for HIV, including MSM (65%) and people born overseas (45%). This is similar to data from public sexual health clinics in NSW in 2022, where 62% of HIV tests were performed among MSM and 41% among people born overseas [24]. It is encouraging that 80% of people with an HIV diagnosis initiated treatment within six months. Although HIV prevalence was lower compared to that found when testing in other services, using DBS in online self-registration may extend the reach of HIV testing to populations who do not regularly attend services and are less engaged in care.

Overall, the proportion of people participating in the NSW DBS Pilot who live outside of major cities (21%) is less than the proportion of all people tested for HCV RNA in NSW who live in rural/regional areas (44%) [25]. Nonetheless, DBS allows people to collect a sample at home or with a clinic on outreach so it may improve uptake among people who are not otherwise able to access a clinic where HCV RNA testing is offered. In the assisted registration pathways, the prevalence of current HCV infection was 17% in community and 14% in prison. This is comparable to Australian studies among people who inject drugs attending drug treatment and needle and syringe programs (17%, 2019–2021) [26] and people in prison (19%, 2014–2019) [27]. The NSW DBS Pilot reached similar populations to Australian studies of HCV but may improve the capacity of sites that do not have access to point-of-care testing, by allowing them to test for HCV with limited resources. Of all HCV treatment initiations in New South Wales for the period 2017–2020 (*n* = 19,351) [28], 2% were diagnosed in the NSW DBS Pilot. The model of high intensity testing campaigns in prison, where large numbers of people can be tested in the yard or other shared space, shows the potential for scale up of DBS in under-resourced settings.

Half of MSM tested for HIV reported no HIV test in the two years prior to enrolment, higher than a survey of MSM in Sydney in 2022 (38% with no HIV test in the last year [29]) and higher than community-based testing sites in 2019 (15% with no HIV test in the last year [30]), demonstrating that the Pilot outperforms other HIV testing strategies to reach people testing less frequently. In a 2016 survey of culturally and linguistically diverse populations in NSW, 80% reported no HIV test in the last year [31] compared to 62% reporting no HIV test in the two years prior to enrolment in the NSW DBS Pilot. Given that the pilot has been successful in reaching people who have not recently tested for HIV, there is an opportunity to sustain engagement with these populations by incorporating evidence-based complementary interventions such as text message reminders [32].

Forty-five percent of all participants reported not having received HCV testing in the two years prior to enrolment. Among people who inject drugs, 32% reported not having received recent HCV testing, lower than a national survey of people who inject drugs (57%) [33], suggesting that the NSW DBS Pilot may be reaching a population of people who inject drugs better linked to healthcare. Promotion of the online self-registration pathway targeted mainly MSM but further investigation is needed to understand how this pathway could enhance HCV testing for other populations.

Although there were 36 community sites participating in the pilot, the median number of tests per site per year enrolled varied widely (IQR 5–46). Further research is needed to identify enablers for testing in sites demonstrating higher testing numbers. The median time from sample collection to arrival in lab was similar across all registration pathways (6–7 days). Reducing the time from collection to arrival in the laboratory could reduce the time to the delivery of results and improve linkage to care, particularly in the community pathway. After the introduction of the visual aid to assist with sample collection, 90% of cards had the three full spots required to process, comparable with an online postal DBS testing project for STIs in England which reported 95% of cards successful processed [34]. The visual aid is an important element in the implementation of any DBS program which requires self-collection.

The findings have critical implications for local, national, and international HIV and HCV testing strategies. The NSW DBS Pilot is ongoing but the possibilities for integration into standard of care are limited by the regulatory environment. HIV testing from DBS sampling is currently approved by the Therapeutic Goods Administration in Australia. However, there are no approved tests for HCV RNA testing outside of a research study, despite DBS being included in WHO recommendations for HCV testing [35], limiting potential integration of DBS into HCV clinical care. Approval would allow DBS to be used as a tool to facilitate HIV and HCV diagnosis outside of a research study and improve access to testing among priority populations and people not recently tested. Alternatively, collecting fingerstick capillary whole-blood samples using microvette sample collection devices for testing in central laboratories could be a viable option in the current regulatory context, and has been used in the UK [36] and Myanmar [37]. Any scale-up of DBS testing should consider support for more frequent high intensity testing campaigns carried out in in prison. The minimal training required to perform DBS makes it a valuable tool to expand the scope of practice in allied health workers including Aboriginal Health Workers and peer workers in harm reduction services. DBS testing via online self-registration is a strategy to improve testing uptake amongst priority populations living in regional and remote areas. Although point-of-care testing for HIV and HCV can allow fast delivery of results to facilitate single visit test-and-treat, there is still a need for a range of testing modalities. Point-of-care HCV RNA testing platforms are expensive and require considerable training due to the increased complexity for operation of point-of-care devices, which may limit scale-up across all services that may be performing testing. DBS allows sample collection with minimal resources (suitable for outreach and to allow people to collect their own sample) and allows sample collection in extreme weather conditions. The latter is important in the Australian context where hot weather can impact functioning of rapid point-of-care testing machines. Further, DBS testing can be combined with point-of-care antibody testing by using it as a reflex testing approach in people who are HCV antibody positive.

There are limitations to this study. Processes for recruitment and treatment initiation were not standardised across pathways and community sites. Some sites provided linkage to care on-site while others referred off-site. Changes to the survey throughout the study period impacted the quality of some variables including recent injecting and recent HCV testing. The lack of control arm is also a limitation. A wider range of approved sample types by the Therapeutic Goods Administration could improve the uptake of HIV and HCV testing but an important element of this is understanding the cost-effectiveness of each modality. An ongoing study will compare testing via DBS sample with other testing modalities to inform decision-making for the implementation of different testing strategies.

## Conclusions

Strategies are needed to improve HIV and HCV testing, to improve treatment initiation, and reach national and international elimination targets. The evaluation of the first four years of the NSW DBS Pilot demonstrates that large-scale DBS sampling is feasible and could improve reach of testing, particularly in prisons and for people who do not frequently test. DBS provides a useful innovation to expand testing for HIV and HCV in Australia and facilitate the goal of ending the HIV and HCV epidemics.

### Supplementary Information

**Additional file 1: Supplementary Figure 1.** Visual aid contained in DBS sampling kit from December 2017. **Supplementary Figure 2.** HIV and HCV DBS screening laboratory testing algorithm. 

## Data Availability

The datasets used and/or analysed during the current study available from the corresponding author on reasonable request.

## References

[1] World Health Organization. Global health sector strategies on, respectively, HIV, viral hepatitis and sexually transmitted infections for the period 2022–2030. Geneva: World Health Organisation; 2022.

[2] Commonwealth of Australia as represented by the department of health. Eighth national HIV strategy 2018–2022. Canberra: Australian Government; 2018.

[3] Australian Government Department of Health (2018). Fifth national hepatitis C strategy.

[4] Witzel TC, Eshun-Wilson I, Jamil MS, Tilouche N, Figueroa C, Johnson CC (2020). Comparing the effects of HIV self-testing to standard HIV testing for key populations: a systematic review and meta-analysis. BMC Med.

[5] Cunningham EB, Wheeler A, Hajarizadeh B, French CE, Roche R, Marshall AD (2022). Interventions to enhance testing, linkage to care, and treatment initiation for hepatitis C virus infection: a systematic review and meta-analysis. Lancet Gastroenterol Hepatol.

[6] Grebely J, Bruneau J, Lazarus JV, Dalgard O, Bruggmann P, Treloar C (2017). Research priorities to achieve universal access to hepatitis C prevention, management and direct-acting antiviral treatment among people who inject drugs. Int J Drug Policy.

[7] Prestage G, Brown G, Keen P (2012). Barriers to HIV testing among Australian gay men. Sex Health.

[8] Easterbrook P, Johnson C, Figueroa C, Baggaley R (2016). HIV and hepatitis testing: global progress, challenges, and future directions. AIDS Rev.

[9] Iversen J, Grebely J, Catlett B, Cunningham P, Dore GJ, Maher L (2016). Estimating the cascade of hepatitis C testing, care and treatment among people who inject drugs in Australia. Int J Drug Policy.

[10] Yehia BR, Schranz AJ, Umscheid CA, Lo Re V (2014). The treatment cascade for chronic hepatitis c virus infection in the united states: a systematic review and meta-analysis. Rizza SA, editor. PLoS One..

[11] Bajis S, Maher L, Treloar C, Hajarizadeh B, Lamoury FMJ, Mowat Y (2018). Acceptability and preferences of point-of-care finger-stick whole-blood and venepuncture hepatitis C virus testing among people who inject drugs in Australia. Int J Drug Policy.

[12] Lim MD (2018). Dried blood spots for global health diagnostics and surveillance: opportunities and challenges. Am J Trop Med Hyg.

[13] Harris M, Rhodes T (2013). Hepatitis C treatment access and uptake for people who inject drugs: a review mapping the role of social factors. Harm Reduct J.

[14] Hickman M, McDonald T, Judd A, Nichols T, Hope V, Skidmore S (2008). Increasing the uptake of hepatitis C virus testing among injecting drug users in specialist drug treatment and prison settings by using dried blood spots for diagnostic testing: a cluster randomized controlled trial. J Viral Hepat.

[15] McLeod A, Weir A, Aitken C, Gunson R, Templeton K, Molyneaux P (2014). Rise in testing and diagnosis associated with Scotland’s action plan on hepatitis C and introduction of dried blood spot testing. J Epidemiol Community Health.

[16] Mohamed Z, Al-Kurdi D, Nelson M, Shimakawa Y, Selvapatt N, Lacey J (2020). Time matters: Point of care screening and streamlined linkage to care dramatically improves hepatitis C treatment uptake in prisoners in England. Int J Drug Policy.

[17] Trickey A, Fajardo E, Alemu D, Artenie AA, Easterbrook P (2023). Impact of hepatitis C virus point-of-care RNA viral load testing compared with laboratory-based testing on uptake of RNA testing and treatment, and turnaround times: a systematic review and meta-analysis. Lancet Gastroenterol Hepatol.

[18] Glasgow RE, Harden SM, Gaglio B, Rabin B, Smith ML, Porter GC (2019). RE-AIM planning and evaluation framework : adapting to new science and practice with a 20-year review. Front Public Health..

[19] Catlett B, Bajis S, Starr M, Dore GJ, Hajarizadeh B, Cunningham PH (2021). Evaluation of the aptima HCV Quant Dx assay for hepatitis C virus RNA detection from fingerstick capillary dried blood spot and Venepuncture-collected samples. J Infect Dis.

[20] Catlett B, Starr M, Machalek DA, Danwilai T, Palmer M, Kelly A (2022). Evaluation of serological assays for SARS-CoV-2 antibody testing from dried blood spots collected from cohorts with prior SARS-CoV-2 infection. J Clin Virol Plus.

[21] Catlett B, Hajarizadeh B, Cunningham E, Wolfson-Stofko B, Wheeler A, Khandaker-Hussain B (2022). Diagnostic accuracy of assays using point-of-care testing or dried blood spot samples for the determination of hepatitis C virus RNA: a systematic review. J Infect Dis.

[22] Catlett B, Carrera A, Starr M, Applegate TL, Lowe P, Grebely J (2019). Performance evaluation of the hologic aptima HCV Quant Dx assay for detection of HCV RNA from dried blood spots. J Clin Virol.

[23] Conway A, Stevens A, Murray C, Prain B, Power C, McNulty A (2023). Hepatitis C treatment uptake following dried blood spot testing for hepatitis C RNA in New South Wales, Australia: the NSW DBS Pilot study. Open Forum Infect Dis.

[24] NSW Health. NSW HIV strategy 2021 – 2025: Quarter 2 data report 2022. Sydney: NSW Health; 2022.

[25] Yousafzai MT, Alavi M, Valerio H, Hajarizadeh B, Grebely J, Dore GJ (2022). Time to hepatitis C RNA testing and treatment in the era of direct-acting antiviral therapy among people with hepatitis C in NSW, Australia. Viruses..

[26] Valerio H, Alavi M, Conway A, Silk D, Treloar C, Martinello M, et al. Declining prevalence of current HCV infection and increased treatment uptake among people who inject drugs: The ETHOS engage study. Int J Drug Policy. 2022;105. Available from: https://linkinghub.elsevier.com/retrieve/pii/S0955395922001256. 10.1016/j.drugpo.2022.103706 35533635

[27] Hajarizadeh B, Grebely J, Byrne M, Marks P, Amin J, McManus H (2021). Evaluation of hepatitis C treatment-as-prevention within Australian prisons (SToP-C): a prospective cohort study. Lancet Gastroenterol Hepatol.

[28] Centre for Population Health, NSW Health (2021). NSW Hepatitis B and Hepatitis C Strategies 2014–2020 Data Report: 2020 Annual Data Report.

[29] Chan, C, Broady, T, MacGibbon, J, Bavinton, B, Mao, L, Molyneux, A, et al. Gay Community Periodic Survey: Sydney 2022. UNSWorks, UNSW;2022. Available from: http://handle.unsw.edu.au/1959.4/unsworks_80836. Cited 2022 Dec 14.

[30] Chan C, Patel P, Johnson K, Vaughan M, Price K, McNulty A, et al. Evaluation of ACON’s community-based a[TEST] HIV and STI testing services, 2015–2019 Internet. UNSWorks, UNSW; 2020. Available from: http://handle.unsw.edu.au/1959.4/unsworks_70527. Cited 2023 Nov 23.

[31] McGregor S, Mlambo E, Gunaratnam P, Wilson D, Guy R (2016). HIV Knowledge, Risk Behaviour and Testing: A community survey in people from culturally and linguistically diverse (CALD) backgrounds in NSW, Australia.

[32] Paschen-Wolff MM, Restar A, Gandhi AD, Serafino S, Sandfort T (2019). A systematic review of interventions that promote frequent HIV testing. AIDS Behav.

[33] Sutherland, R, Karlsson, A, King, C, Jones, F, Uporova, J, Price, O, et al. Australian Drug Trends 2022: Key Findings from the National Illicit Drug Reporting System (IDRS) Interviews. NDARC, Sydney; 2022. Available from: http://handle.unsw.edu.au/1959.4/unsworks_81243. Cited 2022 Oct 25.

[34] Page M, Atabani S, Arumainayagam J, Wilson S, Hartland D, Taylor S (2021). Are all blood-based postal sampling kits the same? A comparative service evaluation of the performance of dried blood spot and mini tube sample collection systems for postal HIV and syphilis testing. Sex Transm Infect.

[35] World Health Organization. Updated recommendations on treatment of adolescents and children with chronic HCV infection, and HCV simplified service delivery and diagnostics. Geneva; 2022. 37410875

[36] NHS England. NHS rolls out order-to-home hepatitis C tests via NHS website for tens of thousands at risk. 2023. Available from: https://www.england.nhs.uk/2023/05/nhs-rolls-out-order-to-home-hepatitis-c-tests-via-nhs-website-for-tens-of-thousands-at-risk/. Cited 2023 May 29.

[37] Nyein P, Tillakeratne S, Phyu S, Yee M, Lwin M, Htike K (2023). Evaluation of simplified HCV diagnostics in HIV/HCV Co-infected patients in Myanmar. Viruses.

