# Supplementary material for: Testing, diagnosis, and treatment following the implementation of a program to provide dried blood spot testing for HIV and hepatitis C infections: the NSW DBS Pilot
Source: BMC Infect Dis. 2024 Jan 29;24:137. doi: 10.1186/s12879-024-08989-8 (PMC10823617; doi:10.1186/s12879-024-08989-8)
Supplement: Supplementary file 1 — Additional file 1: Supplementary Figure 1. Visual aid contained in DBS sampling kit from December 2017. Supplementary Figure 2. HIV and HCV DBS screening laboratory testing algorithm. [file 12879_2024_8989_MOESM1_ESM.pdf]

Supplementary Figure 1: Visual aid contained in DBS sampling kit from December 2017

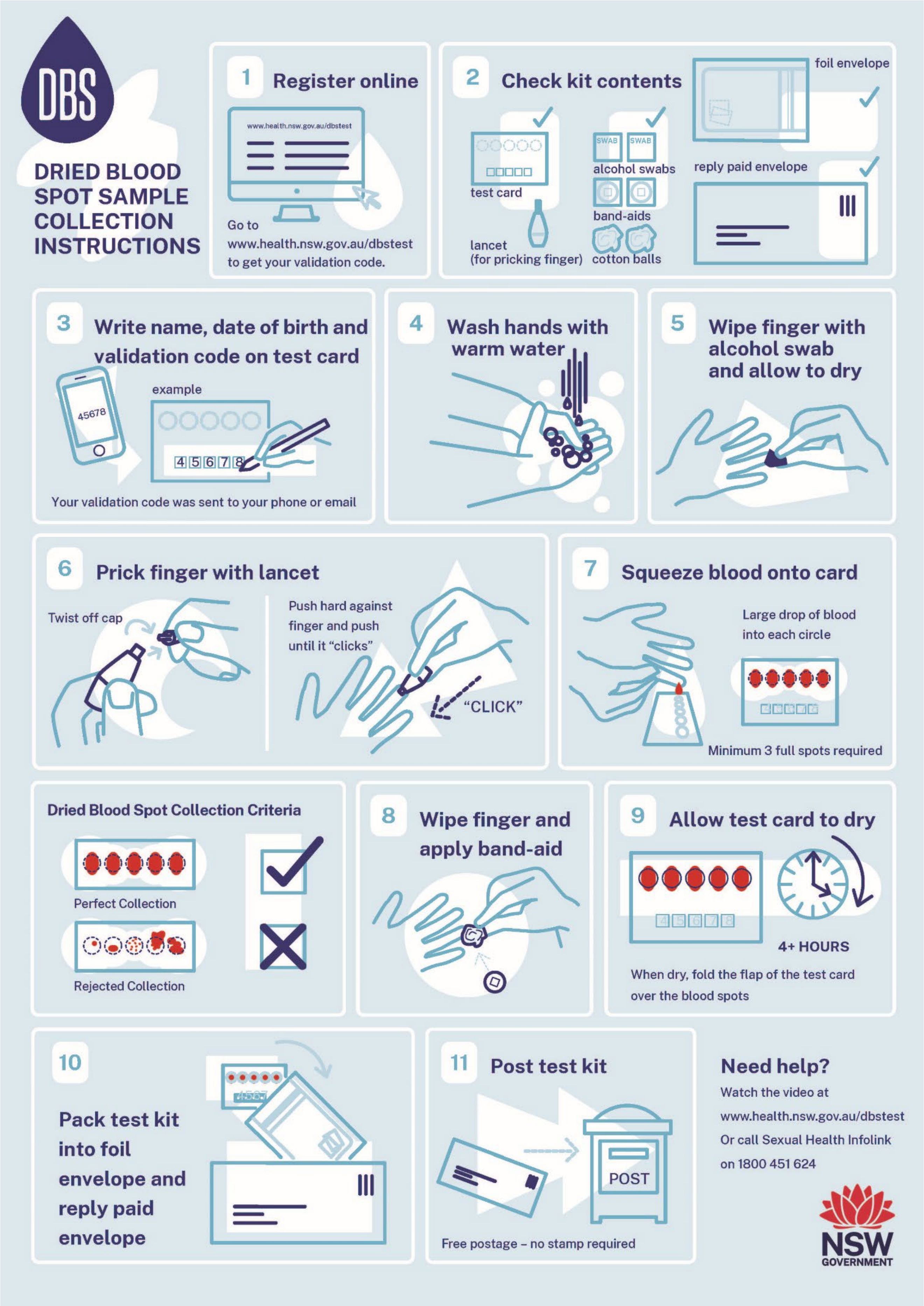

Supplementary Figure 2: HIV and HCV DBS screening laboratory testing algorithm

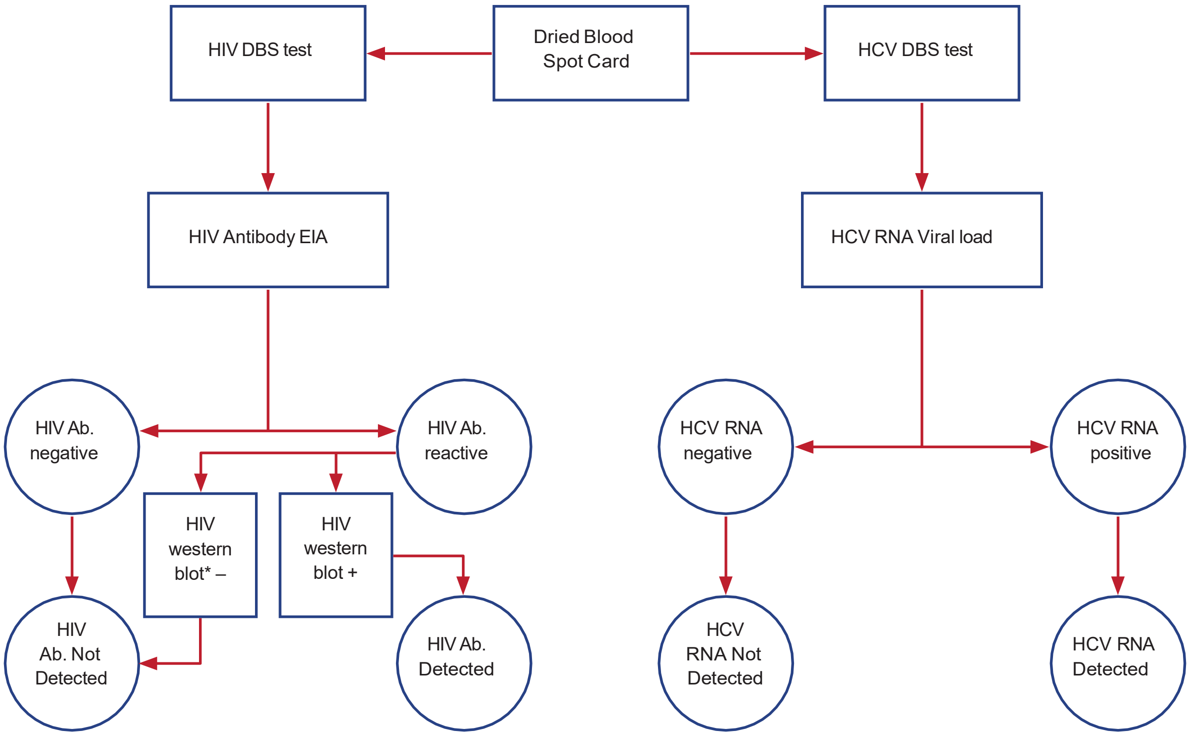

Assay detail:
HIV Ab/Ag EIA: Diasorin Murex HIV Ab/Ag ELISA
HIV Western Blot: Biorad HIV New LAV Blot II
(* indeterminate blot requires conventional venepuncture
HCV RNA viral load: Hologic Aptima HCV Quant Dx (1,2,3)
